# Aberrant expression of STYK1 and E-cadherin confer a poor prognosis for pancreatic cancer patients

**DOI:** 10.18632/oncotarget.22794

**Published:** 2017-11-30

**Authors:** Luguang Chen, Chao Ma, Yun Bian, Chengwei Shao, Tiegong Wang, Jing Li, Xiaodan Chong, Li Su, Jianping Lu

**Affiliations:** ^1^ Department of Radiology, Changhai Hospital of Shanghai, Second Military Medical University, Shanghai, China; ^2^ Cancer Institute, Institute of Translational Medicine, Second Military Medical University, Shanghai, China; ^3^ School of Pharmacy, Second Military Medical University, Shanghai, China

**Keywords:** STYK1, E-cadherin, EMT, pancreatic cancer

## Abstract

Previous studies showed that aberrant Serine/threonine/tyrosine kinase 1 (STYK1, also known as NOK) or/and E-cadherin were involved in the progression of some types of human cancers. However, whether they contributed to the development of pancreatic cancer was unknown. Here, we investigated the prognostic significance of aberrant STYK1 and E-cadherin in pancreatic cancer. Our results showed that STYK1 expression increased while E-cadherin decreased in pancreatic cancer tissues compared with normal pancreas tissues. STYK1 level was positively correlated with lymph node metastasis and clinical stage in pancreatic cancer patients. E-cadherin expression was inversely correlated with STYK1 expression in pancreatic cancer tissue samples. Patients with high STYK1 and low E-cadherin expression had the worst prognosis. In addition, STYK1 knockdown in pancreatic cancer cell lines inhibited cell proliferation, enhanced cell apoptosis, induced cell cycle arrest, and prohibited cell migration, while STYK1 over-expression showed the opposite effects. Silencing STYK1 also increased E-cadherin expression and inhibited epithelial-to-mesenchymal transition (EMT) and p-p38 expression *in vitro*. Over-expression had showed the opposite trends, and treatment with p38 inhibitor, SB203580, could reverse the trends. Thus, STYK1 repressed E-cadherin expression and promoted EMT, mediated by p38 MAPK signaling pathway, which was the possible mechanism for STYK1-mediated pancreatic cancer cell proliferation and migration. In summary, our results showed that STYK1 might be a prognostic marker for pancreatic cancer patients and might be a novel strategy for the treatment of pancreatic cancer.

## INTRODUCTION

Pancreatic cancer, a lethal malignancy worldwide, is estimated that 53,670 new cases of pancreatic cancer patients were diagnosed in the United States in 2017 [1]. Pancreatic cancer progressed extremely fast at the advanced stage. However, it is very hard to detect the disease at the early stage due to the lack of obvious symptoms. Therefore, it is urgently needed to identify specific molecular markers involved in pancreatic cancer that could serve as early diagnostic and prognostic factors, with a hope to discover sensitive target to conquer such an uncontrollable disease.

So far, there are not many sensitive markers and targets for pancreatic cancer and several clinical trials are failed. Only one targeting therapy, erlotinib, which is a tyrosine kinase inhibitor, has met the success, which has been recommended in the National Comprehensive Cancer Network guideline [2]. So we wonder if there is any other gene that belongs to tyrosine kinase or its receptor could work as what erlotinib has done in the clinics. Serine/threonine/tyrosine kinase 1 (STYK1) is a human putative protein kinase that belongs to the receptor protein tyrosine kinase family [3, 4]. STYK1 over-expression has been related to many types of tumors, such as prostate cancer cells [5] leukemia cells [6], breast cancer [7], non-small cell lung cancer [8], ovarian cancer [9], and colorectal cancer [10]. Previous studies have found that STYK1 could promote tumorigenesis through enhancing epithelial-to-mesenchymal transition (EMT) [11]. Epithelial cell marker E-cadherin plays a vital role in EMT procession. Cells with loss of E-cadherin were more responsive to induction of EMT by various growth factors [12]. In this study, we explored the expression of STYK1 and E-cadherin in pancreatic cancer, and analyzed the relationship between their expression levels and clinico-pathological features in the patients with pancreatic cancer. In addition, we verified the oncogenic role of STYK1 by using pancreatic cancer cell lines through regulating p38 MAPK-mediated EMT *in vitro*. These findings proposed a potential prognostic marker and therapeutic target to inhibit pancreatic cancer progression.

## RESULTS

### STYK1 and E-cadherin mRNA expression in pancreatic cancer tissues

**Figure 1:**
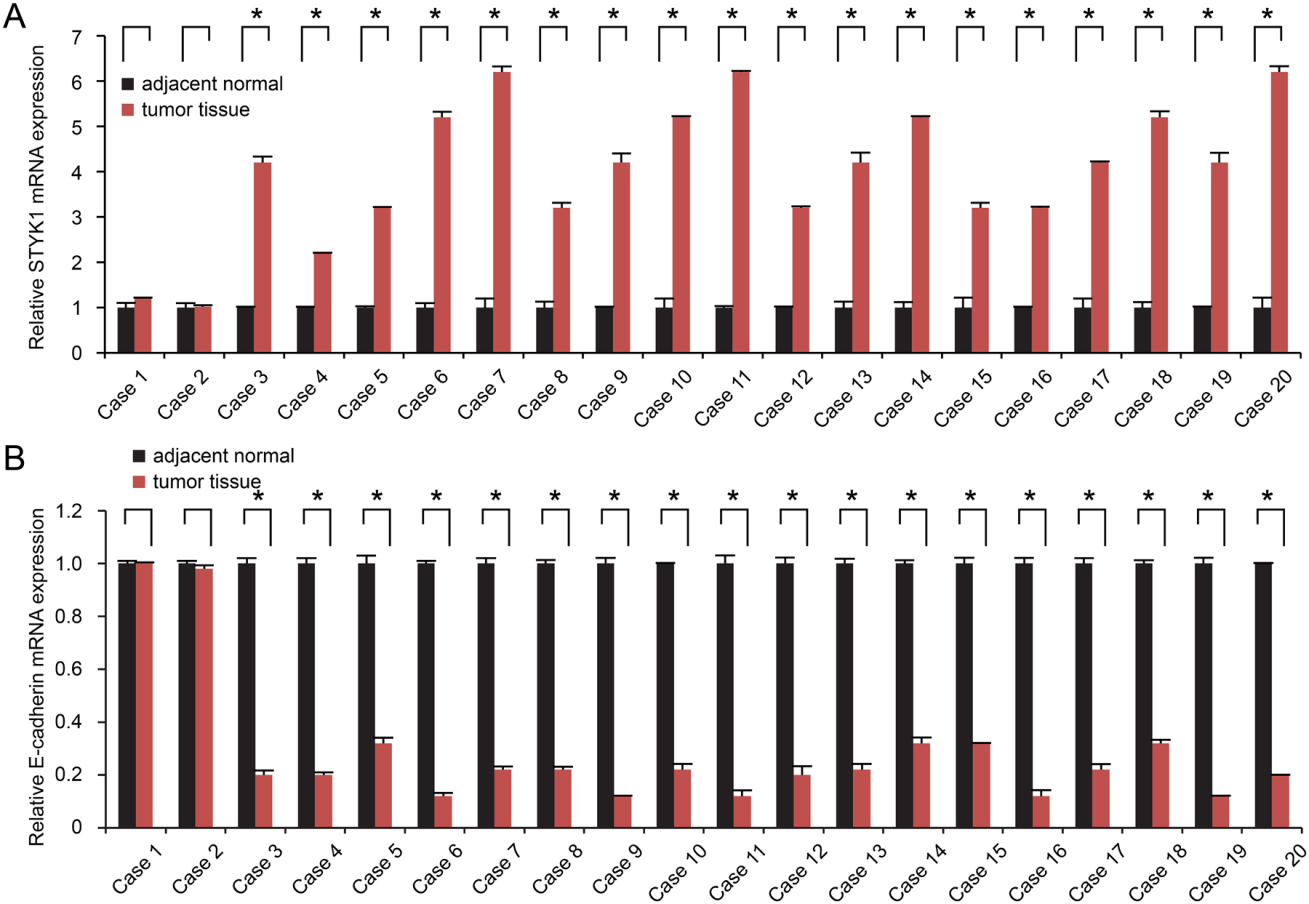
STYK1 and E-cadherin mRNA expression in pancreatic cancer tissues and paired adjacent normal tissues Semi-quantitative RT-PCR was performed to analyze the expression of the STYK1 **(A)** and E-cadherin **(B)** mRNA in 20 cases of pancreatic cancer tissues and adjacent normal tissues. High STYK1 (18/20) and low E-cadherin (18/20) expression were found in pancreatic cancer tissues. Student’s t tests were used.^*^ represented P<0.05, which meant statistically difference.

STYK1 and E-cadherin mRNA expression were detected by semi-quantitative RT-PCR in 20 cases of pancreatic cancer tissue samples and paired adjacent normal tissue samples. Compared with normal tissue samples, STYK1 was highly upregulated in most of pancreatic cancer tissue samples (Figure 1A), whereas E-cadherin mRNA levels were significantly lower in most of tumor samples (Figure 1B).

### Expression of STYK1 and E-cadherin protein level in pancreatic cancer tissues

**Figure 2:**
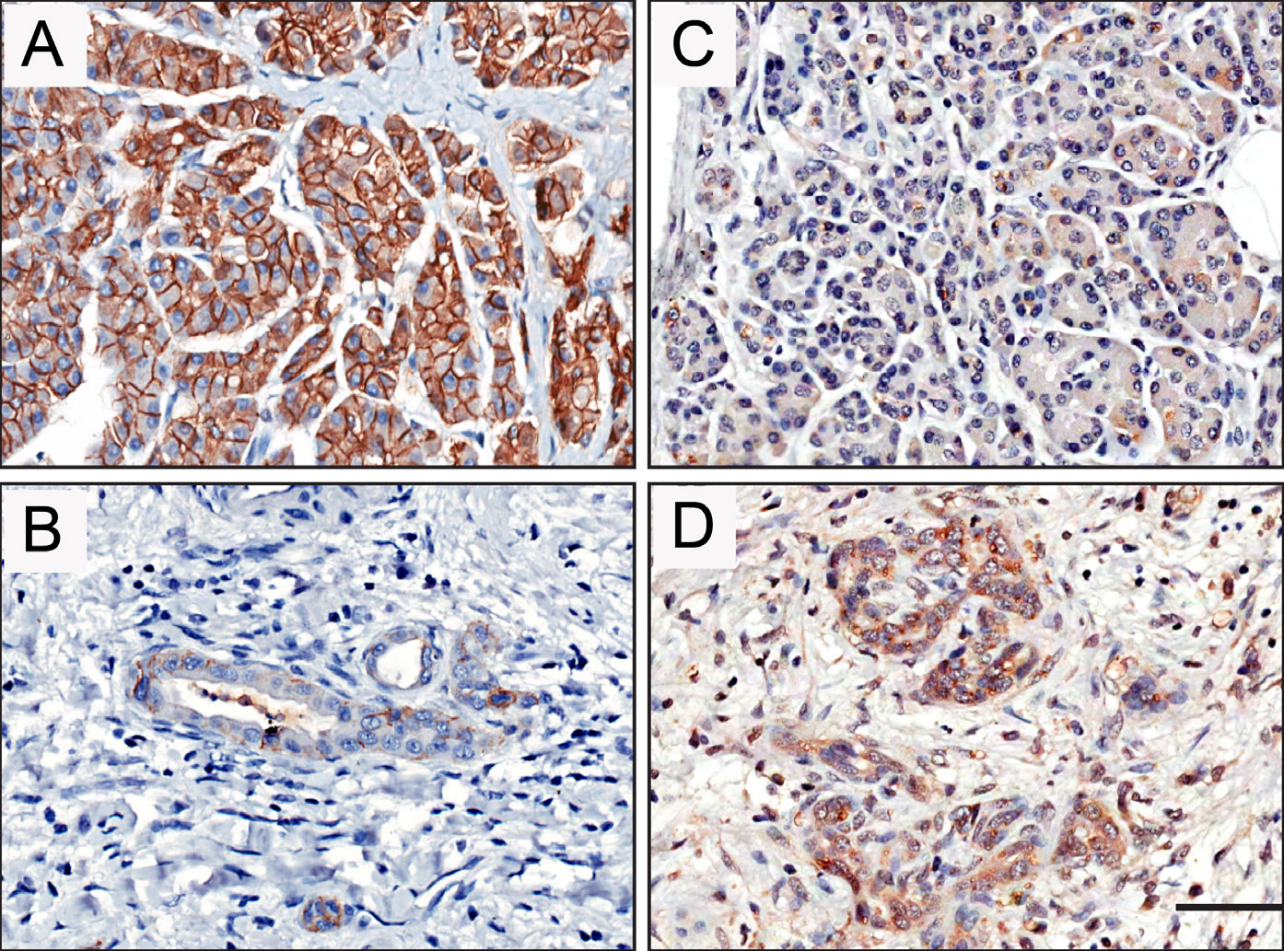
E-cadherin and STYK1 expression levels and localization in pancreatic cancer Immunohistochemistry analysis revealed the STYK1 and E-cadherin expressions. **(A, B)** Strong membrane E-cadherin (A) and low cytoplasmic STYK1 (B) expression were observed in adjacent normal tissues. **(C, D)** Strong cytoplasmic STYK1 (C) and low membrane E-cadherin (D) expression were observed in pancreatic cancer tissues. Scale bar, 50 μm.

We then tried to verify protein levels of STYK1 and E-cadherin by immunohistochemical (IHC) staining. Strong membrane localization of E-cadherin was observed in 87.5% (70/80) of adjacent normal tissues (Figure 2A). In contrast, the expression of E-cadherin was greatly reduced in tumor tissues (Figure 2B). Consistent with mRNA results, STYK1 was expressed at low levels in normal samples (Figure 2C), mainly localized in the cytoplasm. In contrast, high levels of STYK1 were detected in tumor tissues on both cytoplasm and membrane (Figure 2D and Supplementary Figure 1).

Immunohistochemical staining results of 80 samples are summarized in Table 1. Both STYK1 and E-cadherin expression pattern were significantly different between tumor and normal tissue samples (Table 1). These results indicated that STYK1 was upregulated and E-cadherin was downregulated in pancreatic cancer compared with non-tumor tissues.

**Table 1:** Comparisons with E-cadherin and STYK1 expression between pancreatic cancer and paired adjacent normal tissues

Tissue sample	No. of patients	E-cadherin		*P*-value	STYK1		*P*-value
		Low	High		Low	High	
Tumor	80	32	48	0.001^*^	43	37	0.014^*^
Adjacent normal	80	10	70		58	22	

### Relationships of STYK1 and E-cadherin expression with clinico-pathological features

We next analyzed the relationship between E-cadherin and STYK1 expression with clinico-pathological features in patients of pancreatic cancer. STYK1 was positively correlated with lymph node metastasis (P=0.009, Table 2) and clinical stage (P=0.03, Table 2) while other clinico-pathological features such as age, gender, and tumor location were not correlated with its expression. However, the expression of E-cadherin was not correlated with clinico-pathological features in pancreatic cancer. These results suggested that STYK1 may be involved in pancreatic cancer progression.

**Table 2:** Associations between E-cadherin, STYK1 expression and clinico-pathological characteristics in pancreatic cancer

Clinico-pathological parameters	No. of patients	E-cadherin			STYK1		
		Low	High	*P*-value	Low	High	*P*-value
Cases	80	32	48		43	37	
Age (years)							
≤60	37	16	21	0.583^a^	21	16	0.617^a^
>60	43	16	27		22	21	
Gender							
Male	50	23	27	0.157^a^	25	25	0.385^a^
Female	30	9	21		18	12	
Tumor location							
Head, neck	55	23	32	0.622^a^	28	27	0.450^a^
Body, tail	25	9	16		15	10	
Tumor size (cm)							
≤3	25	10	15	1.000^a^	12	13	0.487^a^
>3	55	22	33		31	24	
Tumor differentiation							
Well, moderate	54	24	30	0.242^a^	30	24	0.641^a^
Poor	26	8	18		13	13	
Invasion depth							
T1+T2	68	29	39	0.250^a^	37	31	0.777^a^
T3+T4	12	3	9		6	6	
Lymph nodes metastasis							
N0 (negative)	47	17	30	0.404^a^	31	16	0.009^*a^
N1 (positive)	33	15	18		12	21	
Distant metastasis							
Absent	78	31	47	1.000^b^	42	36	1.000^b^
Present	2	1	1		1	1	
Clinical stage							
Early stages (≤IIa)	45	17	28	0.660^a^	29	16	0.030^*a^
Advanced stages (>IIa)	35	15	20		14	21	

^a^Chi-square test. ^b^Fisher’s exact test. ^*^P<0.05 indicates a significant association among the variables.

### STYK1 and E-cadherin were inversely correlated in pancreatic cancer tissues

**Figure 3:**
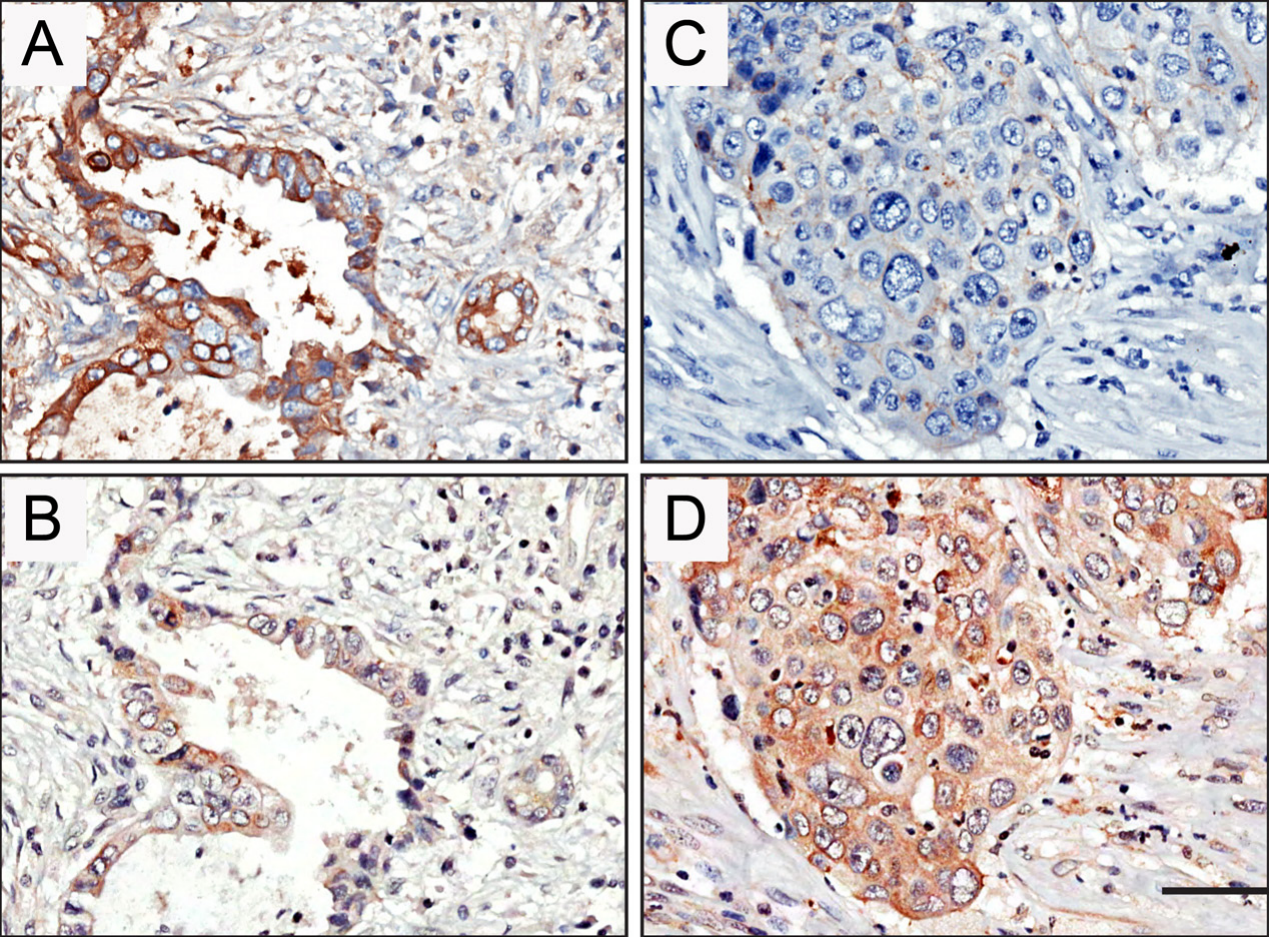
Representative pictures showed negative correlation of STYK1 and E-cadherin expression in pancreatic cancer tissue samples High E-cadherin **(A)** and low STYK1 **(C)** expression were found from one patient with stage I. Low E-cadherin **(B)** and high STYK1 **(D)** expression were detected from another patient with stage IV. Scale bar, 50 μm.

Next, we analyzed correlations between STYK1 and E-cadherin in pancreatic cancers with different clinical stage (Table 3). The representative figures for STYK1 and E-cadherin expression of patients with stage I and stage IV were shown in Figure 3. We found that the STYK1 expression was inversely correlated with E-cadherin (P < 0.001, Table 3 ; Figure 3A-3D).

**Table 3:** Correlation analysis between E-cadherin and STYK1 expression in pancreatic cancer

Tumor tissue sample	STYK1		Correlation coefficient	*P*-value
	Low	High		
E-cadherin Low	9	23	-0.420	<0.001^*^
E-cadherin High	34	14		

### STYK1 and E-cadherin expression were correlated with patient’s prognosis

**Figure 4:**
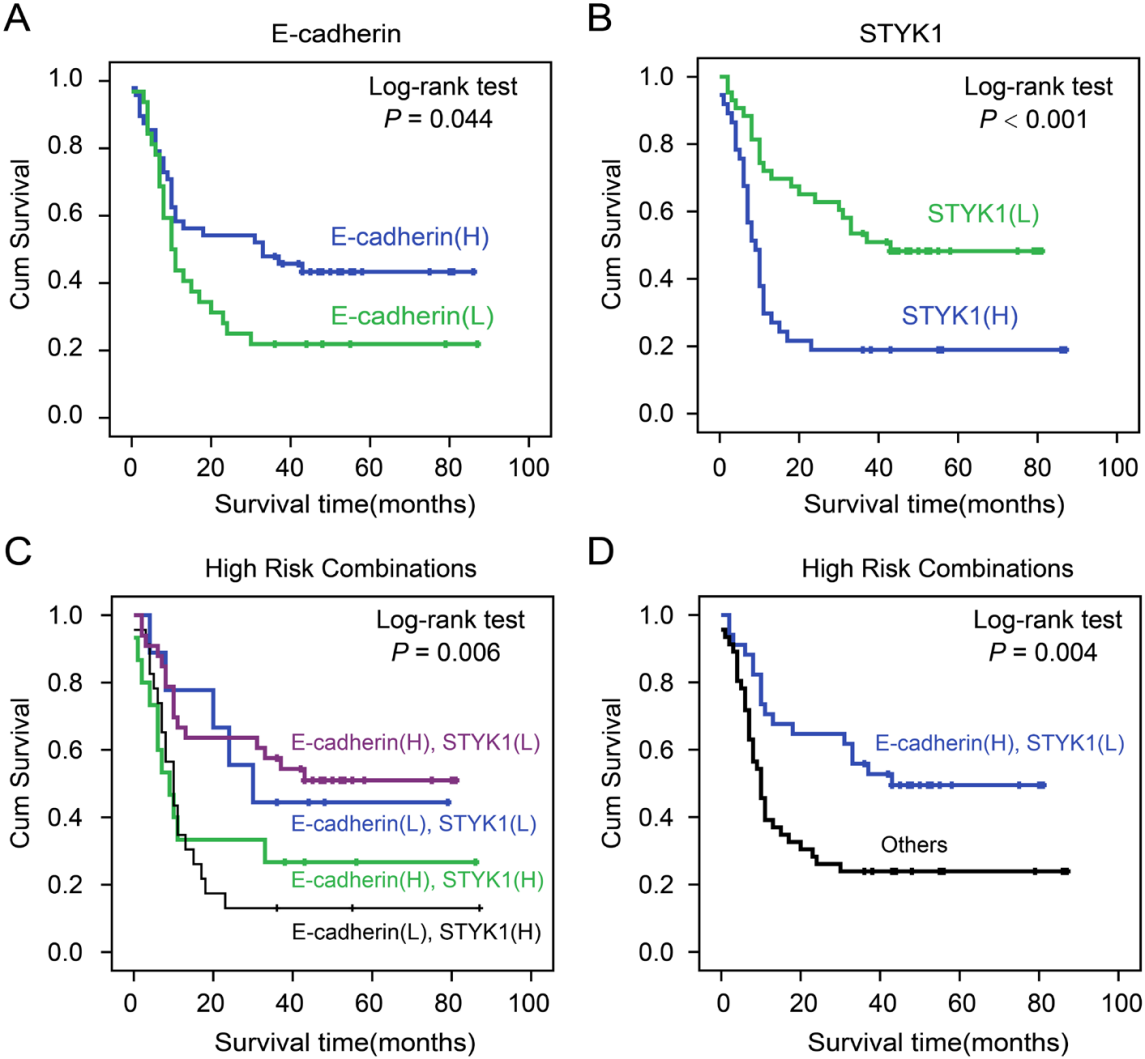
The correlation of STYK1 and E-cadherin expression with pancreatic cancer patients’ prognosis Cumulative Kaplan-Meier overall survival curves of 80 pancreatic cancer patients segmented by E-cadherin **(A)**, STYK1 **(B)**, combination groups (E-cadherin (H), STYK1 (H); E-cadherin (H), STYK1 (L); E-cadherin (L), STYK1 (H); E-cadherin (L), STYK1 (L). **(C** and **D)** Low-risk combination group and others. P-values were calculated by the log-rank test. “L” represents low, “H” represents high.

To further explore the relationship between STYK1 and E-cadherin expression with patient prognosis, a COX regression and Kaplan-Meier analysis were conducted. Univariate survival analysis revealed that, in addition to tumor differentiation, lymph node metastasis, and tumor stage, high STYK1 expression predicted poor prognosis (P<0.05). COX regression model analysis showed that high STYK1 expression was correlated with overall survival (HR=2.191, P=0.008; Table 4). Kaplan-Meier analysis indicated that high E-cadherin and low STYK1 expression in tumor samples favored good clinic outcomes (Figure 4A, 4B). To further investigate the association of survival time with E-cadherin and STYK1 expression, a final concomitant model was constructed and we found that a combinatorial pattern of high E-cadherin expression and low STYK1 predicted the better clinical prognosis in pancreatic cancer (Figure 4C, 4D).

**Table 4:** Summary of univariate and multivariate Cox regression analysis of overall survival duration in all pancreatic cancer patients (n = 80)

Clinico-pathological parameters	Univariate analysis			Multivariate analysis		
	HR	95% CI	*P*-value	HR	95% CI	*P*-value
E-cadhrein						
Low	1					
High	0.579	0.334-1.003	0.051			
STYK1						
Low	1			1		
High	2.689	1.535-4.712	0.001^*^	2.191	1.224-3.923	0.008^*^
Age (years)						
≤60	1					
>60	0.888	0.516-1.531	0.670			
Gender						
Male	1					
Female	0.616	0.341-1.112	0.108			
Tumor location						
Head, neck	1					
Body, tail	1.265	0.741-2.241	0.420			
Tumor size (cm)						
≤3	1					
>3	0.845	0.477-1.497	0.563			
Tumor differentiation						
Well, moderate	1					
Poor	1.881	1.072-3.301	0.028^*^			
Invasion depth						
T1+T2	1					
T3+T4	1.081	0.508-2.300	0.839			
Lymph nodes metastasis						
N0 (negative)	1					
N1 (positive)	1.957	1.131-3.386	0.016^*^			
Distant metastasis						
Absent	1					
Present	2.275	0.550-9.406	0.256			
Clinical stage						
Early stages (≤IIa)	1					
Advanced stages (>IIa)	2.121	1.223-3.676	0.007^*^			

HR hazard ratio, 95% CI 95% confidence interval.

### STYK1 and E-cadherin expression in pancreatic cancer cell lines and normal pancreatic epithelial cell line

**Figure 5:**
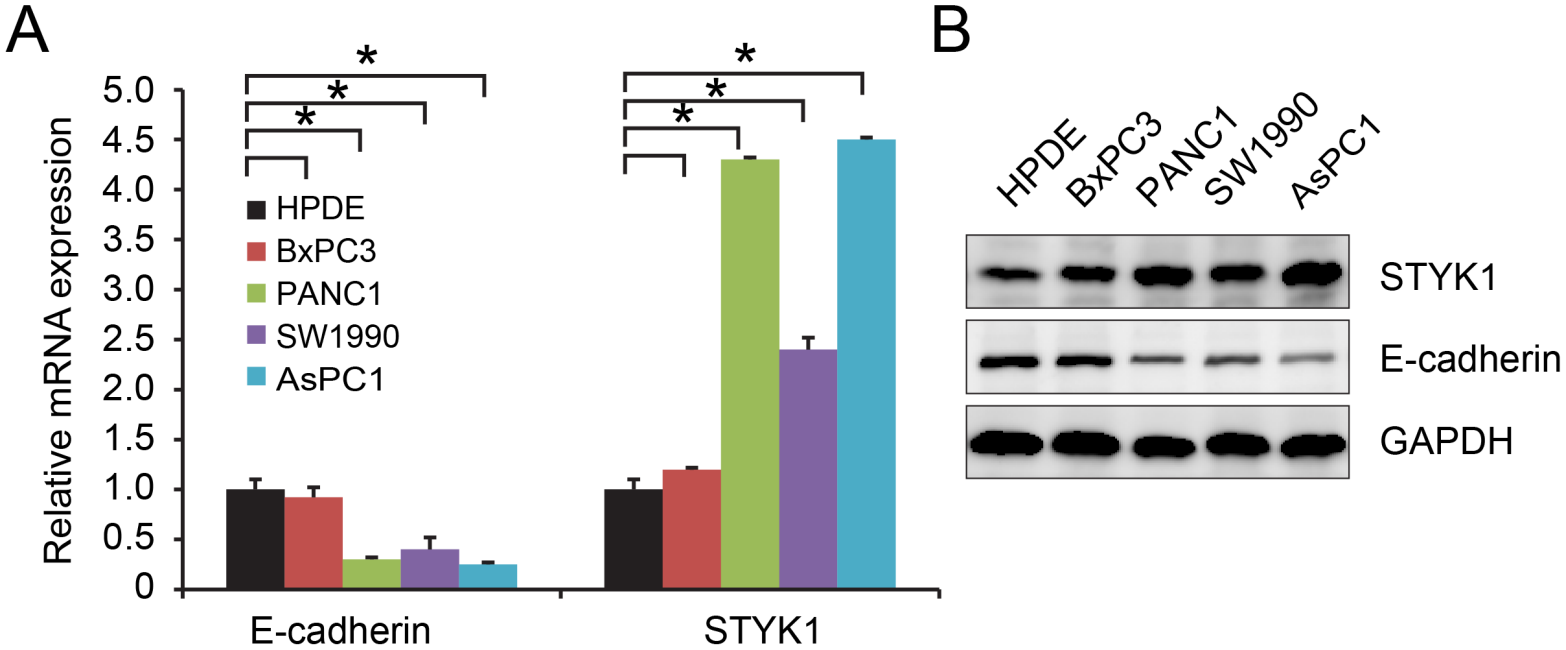
STYK1 and E-cadherin were inversely correlated in pancreatic cancer cell lines **(A)** Higher STYK1 and lower E-cadherin mRNA levels in pancreatic cancer cell lines compared with normal epithelial cell line HPDE. **(B)** Higher STYK1 and lower E-cadherin protein expressions in pancreatic cancer cell lines compared with normal epithelial cell line HPDE.

Finally, we tested the relationship between STYK1 and E-cadherin in human normal pancreatic epithelial cell line HPDE and in pancreatic cancer cell lines *in vitro*. We found that mRNA (Figure 5A) and protein (Figure 5B) expression of STYK1 were higher while mRNA (Figure 5A) and protein (Figure 5B) expression of E-cadherin was lower in pancreatic cancer cell lines compared with HPDE cells. These results suggested that STYK1 might play a key oncogenic role in pancreatic cancer progression and development and might inversely correlated with E-cadherin *in vitro*.

### STYK1 knockdown inhibited cell proliferation and migration through EMT

**Figure 6:**
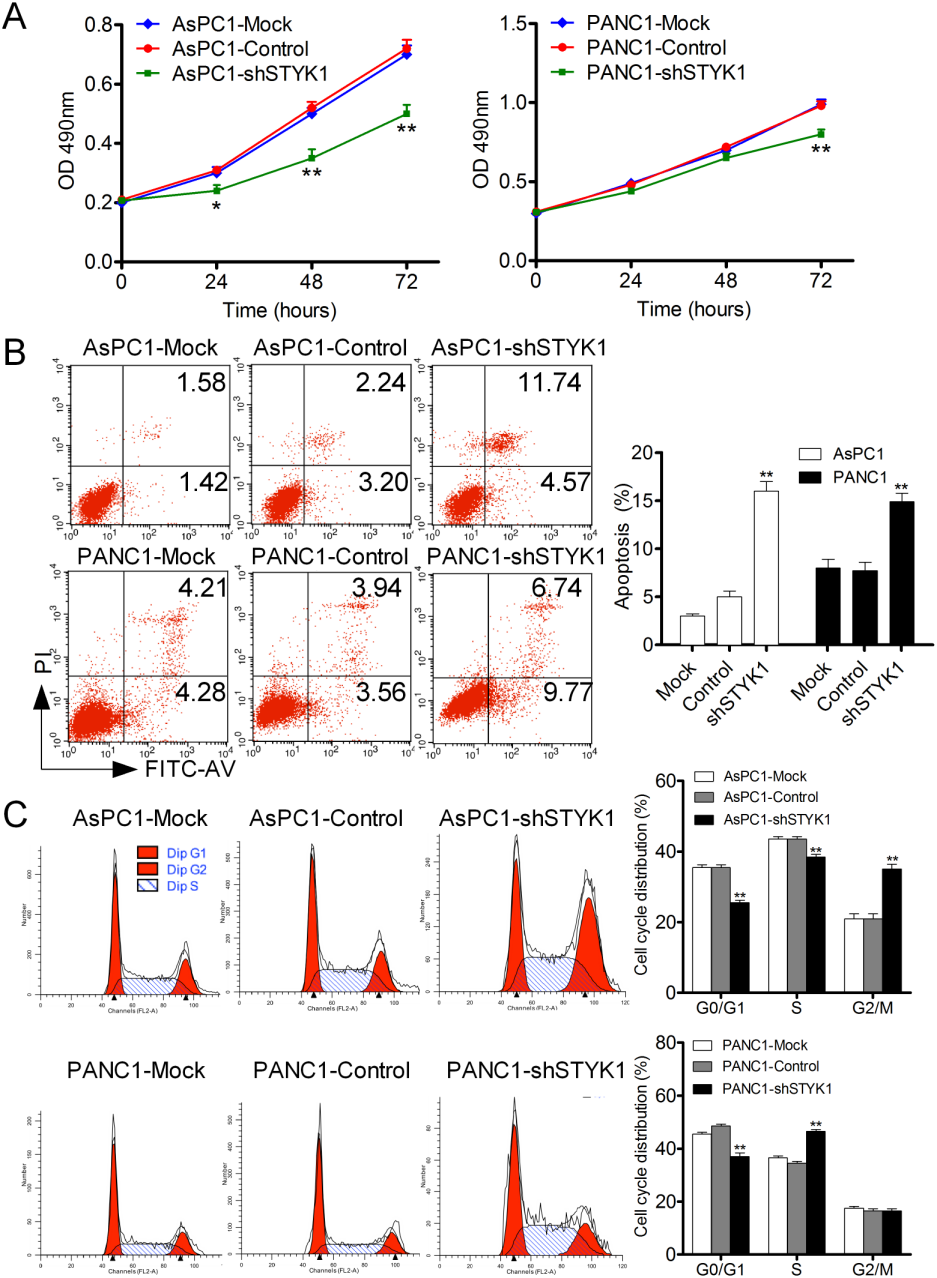
STYK1 knockdown inhibited cell proliferation, promoted apoptosis and induced cell cycle arrest **(A)** AsPC1 and PANC1 cells with shSTYK1 exhibited significantly lower rates of cell proliferation. **(B)** Representative Images of cell apoptosis in mock, control and shRNA-STYK1 groups, demonstrating increased cell apoptosis in cells treated with shRNA-STYK1. **(C)** Representative Images of cell cycle in mock, control and shRNA-STYK1 groups, demonstrating increased cell cycle arrest in cells treated with shRNA-STYK1.

**Figure 7:**
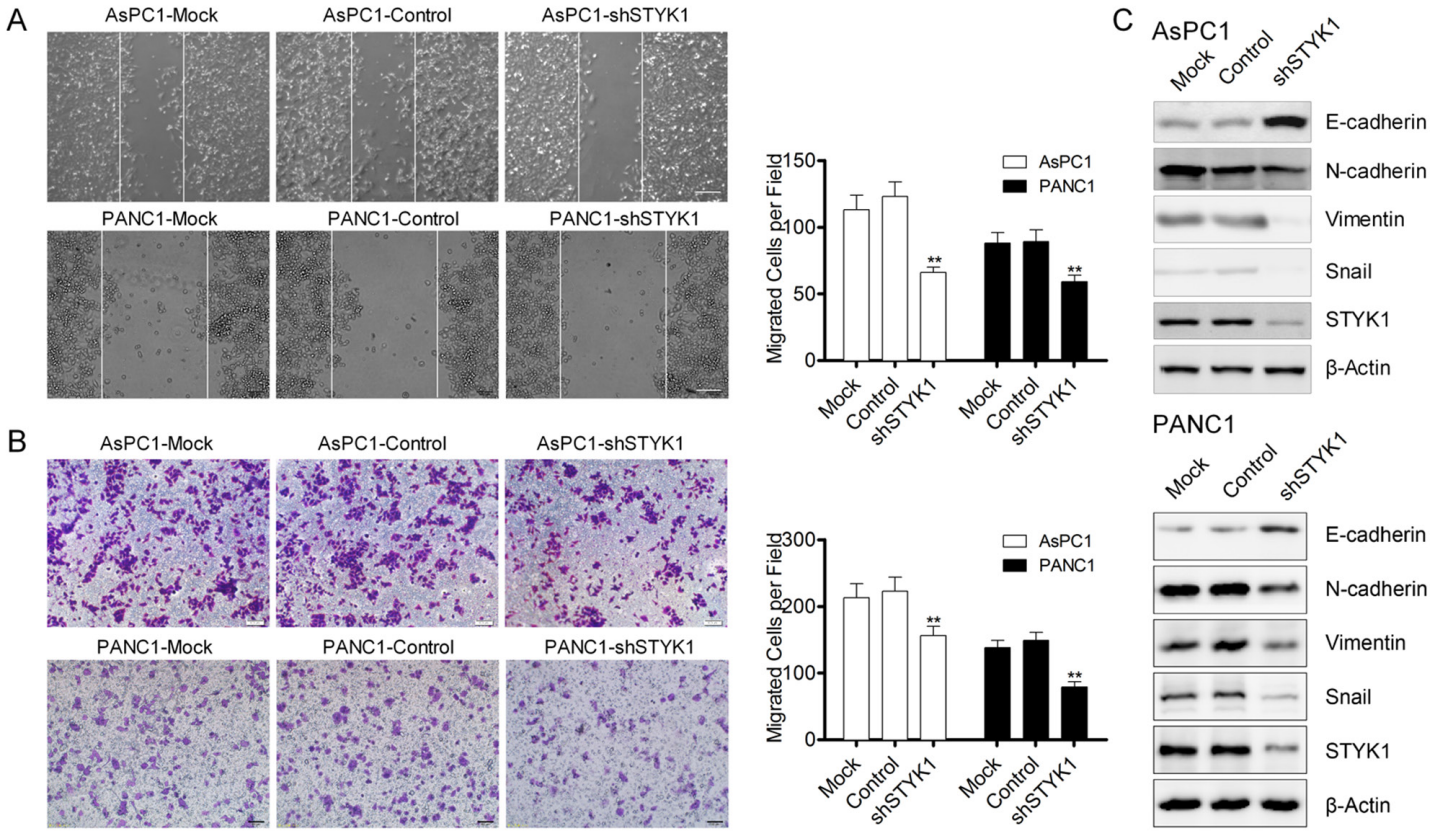
STYK1 knockdown inhibited cells migration through EMT **(A)** Wound-healing assays of ASPC1 and PANC1 cells with Mock, control and shRNA-STYK1, demonstrating a decreased cell migratory ability in shRNA cells. **(B)** Transwell assays of ASPC1 and PANC1 cells with Mock, control and shRNA-STYK1, demonstrating a decreased migrated cell numbers in shRNA cells. **(C)** WB analysis confirmation of STYK1 knockdown efficiency and detect EMT-relative markers in shSTYK1 cells.

We have found that STYK1 was upregulated in pancreatic cancer, and then we would like to explore whether it could alter the biological phenotype of pancreatic cancer. The knockdown efficiency was verified by Western Blot (WB), which showed a strong silencing effect in the shRNA AsPC1 and PANC1 cells (Figure 7C). After STYK1 shRNA transfected, the proliferation rate was lower than that of negative vector determined by SRB assay (Figure 6A). Cell apoptosis was revealed by Annexin V/PI staining in AsPC1 and PANC1 cells and the results maintained that STYK1 knockdown could increase the apoptosis of pancreatic cancer cells (Figure 6B). In addition, knockdown of STYK1 induced an increase the proportion of cells in the proliferation phase (S+G2/M) compared to the control (Figure 6C). Moreover, compared with control cells, decreased cell migration was observed in STYK1-depleted ASPC1 and PANC1 cells (Figure 7A, 7B). Previous studies reported that cell proliferation and migration were associated with EMT [13]. We then tried to explore whether STYK1 functioned through EMT. Our results showed that STYK1 knockdown upregulated the expression of epithelial marker E-cadherin while it downregulated mesenchymal protein markers (Figure 7C).

### P38 MAPK pathway blocking reversed STYK1-mediated EMT, inhibiting cell proliferation and migration

**Figure 8:**
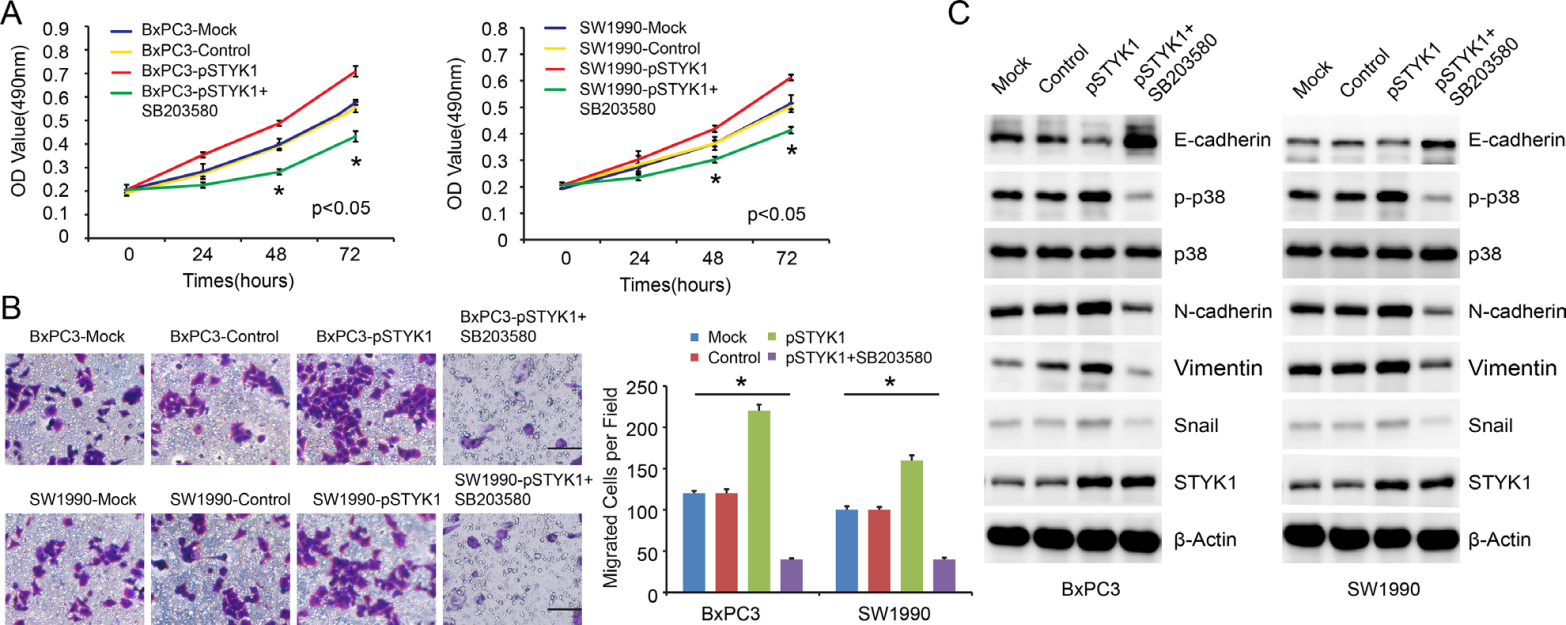
STYK1 promoted pancreatic cancer cell proliferation and migration through p38 MAPK signaling pathway **(A)** Cells with pSTYK1 exhibited significantly higher rates of cell proliferation, and SB203580 reversed this effect. **(B)** Representative images and counted numbers of migrated cells, demonstrating increased migrated cells in pSTYK1 cells, and decreased cells in SB203580 treated groups. **(C)** WB analysis of EMT-relevant markers and p38 MAPK pathway, demonstrating that STYK1 mediated EMT through p38 MAPK pathways. Scale bar, 100 μm.

Moreover, we detected the over-expression effects on BxPC3 and SW1990 cells. The results showed that over-expression of STYK1 could obviously promote cell proliferation (Figure 8A) and migration (Figure 8B). WB showed that STYK1 over-expression downregulated the expression of epithelial marker E-cadherin while it upregulated mesenchymal protein markers (Figure 8C). Previous studies showed that p38 MAPK signaling pathway was correlated with EMT and involved in cancer progression [14, 15]. We next focused on the p38 MAPK pathways. We could see that p-p38 was upregulated in over-expression group, which implied that this pathway had been activated. Next, we used p38 inhibitor, SB203580, to detect whether this inhibitor could reverse this effect. In our results, these enhancing effects, such as rapid increasing proliferation and migration rate, were disappeared with the p38 inhibitor SB203580 added (Figure 8A-8C). Our results indicated that STYK1 promoted pancreatic cancer progression, possibly through p38 MAPK-mediated EMT procession.

## DISCUSSION

Receptor protein tyrosine kinase have been implicated in the regulation of a variety of cellular processes including cell proliferation, apoptosis, and migration through various mechanisms of action and most of receptor protein tyrosine kinase are considered as oncogenes [16, 17]. STYK1, as a member of receptor protein tyrosine kinase, was also shown to be oncogenic as overexpression of STYK1 promoted tumorigenesis and metastasis [3, 5]. In addition, high levels of STYK1 were associated with enhanced EMT. E-cadherin is reported that it is critical for epithelial cells and dysfunctions of the E-cadherin play an important role in pancreatic tumor progression [18, 19]. However, little is known about its expression and the relevant functions in pancreatic cancer. In this study, we focused on STYK1 and E-cadherin expression and their correlations in pancreatic cancers patients and its possible mechanisms.

We examined STYK1 and E-cadherin mRNA and protein expression in pancreatic cancer tissues and corresponding adjacent non-cancerous tissues, and we found that strong STYK1 and weak E-cadherin expression in tumor tissues samples compared with normal tissue samples. This is consistent with studies on other types of cancer, such as glioma [20], cholangiocarcinoma [21], and colorectal cancer [10]. Immunohistochemical analysis of 80 paired pancreatic cancer specimens revealed that STYK1 was mainly localized in the cytoplasm, and 87.5% (70/80) of the tumor tissues tested were positive staining. E-cadherin is localized in cell membrane, and mostly strong stained in normal tissue samples. Several previous studies reported that STYK1 was over-expressed in several tumors [5, 9, 10]. The significance and prognostic value of STYK1 were also demonstrated in other types of cancers. Not only mRNA level [22] of STYK1, but also protein expressions [10] could be also a potential marker to predict the therapy of malignancies. However, there was no clinical investigation of STYK1 on pancreatic cancer. Our research was to analyze the clinical significance of STYK1 and showed that over-expression of STYK1 protein was significantly correlated with clinical stage and lymph node metastasis, indicating that STYK1 might be involved in the progression of pancreatic cancer. However, E-cadherin expression was not correlated with any clinical features. Likewise, Chen P *et al.* also demonstrated a significant correlation of STYK1 protein expression with the grade of tumor differentiation, TNM stage and lymphatic metastasis in non-small cell lung cancer [23]. However, an earlier study conducted by Amachika T *et al.* showed that there were no obvious correlations between STYK1 mRNA expression and clinicopathologic features of patients with lung cancer [8]. Orang AV *et al.* reported that the high expression of STYK1 mRNA in CRC was only correlated with the increased tumor size in their cohort [24]. Therefore, these discrepancies may be due to the different type of studies and samples that used in the experiment. E-cadherin is a critical marker in EMT procession and its expression is correlated with many genetic factors, not only influenced by STYK1. Besides, only 80 cases were analyzed, some bias might be existed in our samples which might lead to such results. In the future, we will enroll more samples to verify the results, largely minimizing the bias.

We then went further to explore whether STYK1 could change the phenotype of pancreatic cancers. We found that silencing STYK1 increases E-cadherin expression, whereas decreased mesenchymal markers, which was consistent with previous research in hepatocellular carcinoma [25]. Cell proliferation and migration were blocked when STYK1 was silenced. These results strongly supported that STYK1 could promote EMT as reported previously in liver cancer [11]. Accumulating data indicated that EMT results in increasing cell migration in various types of cancers [13, 26, 27]. The p38 MAPK signaling pathway was correlated with EMT and involved in cancer progression [14, 15]. We then intended to investigate whether p38 pathway could be the possible mechanism for STYK1-mediated EMT. We constructed over-expression plasmid and performed the functional experiments, and consistently over-expression did the opposite effects. However, after we used p38 MAPK inhibitor, SB203580, the previous enhanced proliferation and migration were prohibited, and reversed the STYK1-mediated EMT-relevant proteins. These results implied that STYK1 promoted pancreatic cancer cell progression through p38 MAPK-mediated EMT signaling pathway. As for the other possible mechanisms, a previous study showed that STYK1 interacted and formed complexes with both Akt and GSK-3β [28]. Another research showed that STYK1 overexpression could enhance the phosphorylated ERK and AKT [3]. Besides, STYK1-mediated acceleration of tumor cell migration and EMT were dependent on MEK/ERK and PI3K/AKT signaling [11, 25]. Activation of MEK/ERK and PI3K/AKT contributes to cell growth [29, 30], promotes migration and EMT [31, 32]. Given the importance of the PI3K, Akt and GSK-3beta signalings in the cell migration, the possible mechanism of STYK1 regulation of E-cadherin, in part, might be through PI3K, Akt and GSK-3beta signaling pathways, which accounted for the oncogenic property of STYK1 protein. There are many crosstalks between pathways, and the mechanism could not be only one pathway participation. Here, we specifically explored p38 MAPK pathway, and further crosstalk investigations should be investigated later. Taken together, the present study indicated that STYK1 promotes pancreatic cancer cell proliferation and migration through enhancing EMT that mediated by p38 MAPK signaling pathway.

In conclusion, the present study demonstrated that STYK1 was highly expressed pancreatic tumor tissues and was significantly correlated with the prognosis of pancreatic cancer. STYK1 repressed E-cadherin expression and promoted EMT, mediated by p38 MAPK signaling pathway, which was the possible mechanism for STYK1-mediated pancreatic cancer cell proliferation and migration.

## MATERIALS AND METHODS

### Tissue samples and immunohistochemistry

80 pancreatic cancer tissue samples and corresponding non-tumor tissues were collected from our hospital. The samples contained well-documented clinico-pathological information, including patients’ age, gender, tumor size and location, tumor differentiation, invasion depth, lymph node metastasis, distant metastasis, tumor stage and follow-up data. Written informed consent was obtained from all participants and the present study was approved by the Ethics Committee of Changhai Hospital.

Pancreatic cancer tissue samples were fixed in 4% paraformaldehyde, dehydrated for 12 h, embedded in paraffin wax, cut into 3 μm-thick slices, and then incubated with the antibody against STYK1 (ab97451, dilution 1:100; Abcam, Cambridge, MA, USA) or E-cadherin (No. 562869, dilution 1:100; Becton, Dickinson and Company, USA) at 4°C overnight and then with horseradish peroxidase (HRP) (Gene Tech GTVision III Detection Kit, Shanghai, China) at room temperature for 40 min. Following 3 washes with PBS, the signal was detected with 3, 3’-diaminobenzidine (DAB) solution.

### Scoring of immunohistochemistry

Five visual fields from different areas of each specimen were chosen at random for the immunohistochemistry evaluation. STYK1 and E-cadherin expression were scored according to staining intensity and the percentage of positive cells as previously described [33]. The percentage of positive cells was scored as follows: 0 score, no positive cells; 1 score, ≤10% positive cells; 2 score, 10–50% positive cells; 3 score, >50% positive cells. Staining intensity was scored as follows: 0 score, no staining; 1 score, faint staining; 2 score, moderate staining; 3 score, dark staining. Comprehensive score = staining percentage × intensity. E-cadherin expression was classified as follows: ≤4 score, low expression; >4 score high expression. STYK1 expression: < 2 score, low expression; ≥ 2 score, high expression.

### Cell lines, human samples, and reagents

Normal human pancreatic ductal epithelial cell line HPDE and pancreatic cancer cells lines, including PANC-1, AsPC-1, SW1990, and BxPC-3, were all obtained and tested authentication from the Chinese Academy of Sciences Cell Bank with 5 passages (www.cellbank.org.cn, Shanghai, China). PANC-1 cells were cultured in DMEM supplemented with 10% fetal bovine serum (FBS) (both from Gibco, USA) in 5% CO_2_ saturated humidity at 37°C. HPDE, BxPC-3, and AsPC-1 cells were cultured in RPMI 1640 (Gibco) supplemented with 10% FBS under 5% CO2 saturated humidity at 37°C. SW1990 cells were cultured in L-15 medium (Gibco) supplemented with 10% FBS and grown in full air condition at temperature of 37°C. The p38 MAPK inhibitor, SB203580 was bought from Selleck.

### Establishment of knockdown and over-expression cell lines

Cells were transfected with 1.6 μg shSTYK1 in the presence of 4 μl lipo2000 in 6-wells plates according to manufactures’ instructions. shRNAs for STYK1 and one negative control shRNA were designed and synthesized by Genepharm Technologies (Shanghai, China). The STYK1 shRNA sequences were as follows: 5′-CCGGGAAGCAGTATGAAGTGATTATCTCGAGATAATCACTTCATACTGCTTCTTTTTTG-3′. The coding sequence fragments of STYK1 gene were obtained by PCR using the following primers: forward, 5′-AAATCTAGAATGGGCATGACACGGATG-3′, 5′-AAAGCGGCCGCTCAAAGCATGCTATAGTTGTAGAAG-3′. The plasmid was also conducted by the Genepharm Technologies (Shanghai, China). The over-expression plasmids were transfected into BxPC3 and SW1990. Transfection effects were testified by WB at 48 hours of post-transfection.

### RNA isolation and semi-quantitative RT-PCR analysis

We extracted total RNA in cultured cells using RNA extraction kit (Takara Bio, Inc.). Then, we performed the Reverse transcription and Semi-quantitative RT-PCR process to evaluate expression of STYK1. The PCR program included 1 cycle of 95°C for 30 s, 40 cycles of 95°C for 5 s and 60°C for 30 s. GAPDH expression was used to normalize for variance. The PCR Primers pairs used for each genes were as follows:

STYK1 sense, 5′-AAATCTAGAATGGGCATGACACGGATG-3′;

STYK1 antisense, 5′-AAAGCGGCCGCTCAAAGCATGCTATAGTTGTAGAAG-3′;

GAPDH, sense, 5′-CCCCGCTACTCCTCCTCCTAAG-3′;

GAPDH antisense, 5′-TCCACGACCAGTTGTCCATTCC-3′;

### Western blot analysis

The prepared cells were lysed using RIPA (Beyotime, shanghai, China) and the protein concentration was determined by a Bradford kit (Beyotime). We first fractionated the total proteins (20 μg) by SDS-PAGE and then the gels were transferred onto a polyvinylidene fluoride (PVDF) membrane using bio-rad trans-blot. Then, we block the membranes using 3% bovine serum albumin (BSA) for 1h and then indicated primary antibodies were incubated at 4°C overnight. And next, we incubated secondary antibodies for 1h at room temperature and finally, enhanced chemiluminescence detection system was used to detect the protein expression. The interior criterion for expression was using β-Actin or GAPDH as a loading control. The antibodies used here were: anti-E-cadherin; anti-STYK1, anti-N-cadherin (#13116, 1:1000, Cell Signaling Technology), anti-Snail (#3879, 1:1000, Cell Signaling Technology), anti-p38 and anti-p-p38 (#8690 and #9216, 1:1000, Cell Signaling Technology), anti-GAPDH (#5174, 1:2000, Cell Signaling Technology), anti-Vimentin (#5741, 1:1000, Cell Signaling Technology), and anti-β-Actin (sc-7210, Santa Cruz Biotechnology, Inc., CA, USA).

### Cell proliferation, apoptosis and cell cycle assay

Cells were transfected with STYK1-nc and STYK1-sh and cultured for 24, 48, 72 h, respectively. Then, the cells were fixed with 100 μl 10% trichloroacetic acid for 30 min at 4°C. 100 μl SRB solutions (in 0.4% w/v in acetic acid) were put in each sample for staining at room temperature for 20 min. After that, the plates were washed with 200 μl 1% acetic acid. Optical density (OD) values were measured at 540 nm with a reference wavelength of 630 nm by microtiter plate reader (VERSMax) after adding the 100 μl 10 mM Tris-base (pH 10) for solubilization.

Cells were plated into 6-well plates at 3 × 105 per well for cell cycle and apoptosis analyses. After treatment, cells were harvested and washed twice with pre-cooled PBS for Cell Cycle analysis using Cell Cycle Staining Kit (MULTI SCIENCES #CCS012) and apoptosis analyses using Annexin V-FITC/PI apoptosis kit (MULTI SCIENCES# AP101-100-kit) according to the manufactures’ protocol by FACSCaliburTM flow cytometry (BD).

### Wound healing and cell migration assays

For the measurement of cell migration, cells were kept in a serum-free medium for 24 hours and wounded with a plastic micropipette tip with a large orifice. The wounded monolayers were rinsed with PBS, and then incubated with basal medium. The images after wounding were recorded in five random fields by using an inverted microscope (Leica DMI 3000B, Wetzlar, Germany) both before and after a 24 h treatment at the same position.

Cell migration assays were conducted using specialized MilliCell chambers (Millipore, Bedford, USA). We inserted an 8 μm pore size polycarbonate membrane in the plate, and placed 10% FBS medium in the lower chambers to serve as a chemo-attractant. 1×10^5^ AsPC-1 or PANC-1 cells harvested in a 100 μl FBS-free medium were placed in the upper chambers and incubated at 37°C for less than 24 h. Migrated cells on the other side of membrane surface were stained by 0.1% Crystal Violet Staining Solution for 15 min. Scraping off the upper surface cells with cotton swabs and counted five selected fields under a microscope at a ×200 magnification.

### Statistical analyses

Data are calculated as the mean ± SD using SPSS 17.0. Values and percentages between groups were compared using Student’s t tests and chi-square tests, respectively. We analyzed the correlation of STYK1 or E-cadherin expression with clinical characteristics with χ2 tests or Fisher’s exact methods. Overall survival is assessed using a Kaplan-Meier method and univariate and COX regression analysis. Those parameters with a P value <0.05 in the univariate analyses were calculated in a COX regression model. All P-values were two-sided and the P value < 0.05 is considered significant.

## SUPPLEMENTARY MATERIALS FIGURE


