# Supplementary material for: Aberrant expression of STYK1 and E-cadherin confer a poor prognosis for pancreatic cancer patients
Source: Oncotarget. 2017 Nov 30;8(67):111333–45. doi: 10.18632/oncotarget.22794 (PMC5762325; doi:10.18632/oncotarget.22794)
Supplement: Supplementary file 1 [file oncotarget-08-111333-s001.pdf]

Aberrant expression of STYK1 and E-cadherin confer a poor prognosis for pancreatic cancer patients

SUPPLEMENTARY MATERIALS

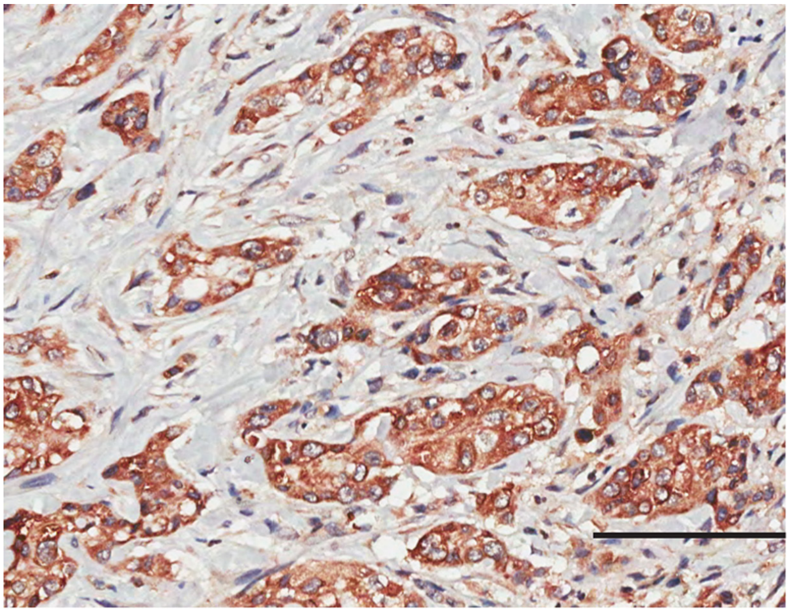

Supplementary Figure 1: Representative pictures showed STYK1 localization in pancreatic cancer tissue. STYK1 expression was mainly localized in the cytoplasm, and weak staining in the membrane. Scale bar, 100 µm.
